# Application of Capillary Electrophoresis to the Analysis of Bioactive Compounds in Herbal Raw Materials

**DOI:** 10.3390/molecules26082135

**Published:** 2021-04-08

**Authors:** Anna Przybylska, Marcin Gackowski, Marcin Koba

**Affiliations:** Department of Toxicology and Bromatology, Faculty of Pharmacy, L. Rydygier Collegium Medicum in Bydgoszcz, Nicolaus Copernicus University in Torun, A. Jurasza 2 Street, PL-85089 Bydgoszcz, Poland; marcin.gackowski@cm.umk.pl (M.G.); kobamar@cm.umk.pl (M.K.)

**Keywords:** capillary electrophoresis, herbal, raw material, tea, polyphenols, flavonoids, amino acids, coumarins, alkaloids

## Abstract

The article is a summary of scientific reports from the last 16 years (2005–2021) on the use of capillary electrophoresis to analyze polyphenolic compounds, coumarins, amino acids, and alkaloids in teas or different parts of plants used to prepare aqueous infusions, commonly known as “tea” or decoctions. This literature review is based on PRISMA guidelines and articles selected in base of criteria carried out using PICOS (Population, Intervention, Comparison, Outcome, Study type). The analysis showed that over 60% of articles included in this manuscript comes from China. The literature review shows that for the selective electrophoretic separation of polyphenolic and flavonoid compounds, the most frequently used capillary electromigration technique is capillary electrophoresis with ultraviolet detection. Nevertheless, the use of capillary electrophoresis-mass spectrometry allows for the sensitive determination of analytes with a lower limit of detection and gives hope for routine use in the analysis of functional foods. Moreover, using the modifications in electrochemical techniques allows methods sensitivity reduction along with the reduction of analysis time.

## 1. Introduction

Is common use, the word “tea” means an aqueous infusion prepared from dried herbal materials. It is one of the most popular beverages in the world, which is made from a variety of plants, other than *Camellia sinensis*. Real tea is prepared from leaves and buds of *Camellia sinensis.* It is estimated that the highest consumption of tea is in China [1].

Tea, especially green tea, is a rich composition of compounds that prevent obesity and exhibit anti-cancer, anti-diabetic properties, and reduces the risk of cardiovascular problems [2,3,4]. *Evodiae Fructus* has been used in traditional Chinese medicine for over 2000 years, and the limonin as a major metabolite responsible for anti-HIV effects [5]. Tea is also a part of the Mediterranean diet used by Italians and Greeks, which may contribute to the prevention of chronic diseases such as metabolic syndrome and obesity [3]. Moreover, studies have shown that long-term consumption of polyphenols can reduce chronic inflammation and oxidative stress and at the same time inhibit the growth, reproduction, and diffusion of cancer cells. Meta-analysis found that green tea consumption potentially reduced cancer risk by 15% [6]. The rich properties of teas come from profile of biologically active compounds. Additionally, teas and aqueous infusions are rich in catechins, epicatechins, and epicatechin gallate and epigallocatechin gallate amino acids and organic acids [3,7,8]. The production process of herbal raw materials is not standardized, which means that the profile of compounds present in leaves or inflorescences is different and depends on the fermentation processes [9]. Studies with Chinese black, green, and blue (oolong tea) teas indicate that black tea is the richest in gallic acid (582.43 µg g^−1^), vanillic acid (4.31 µg g^−1^), trans-p-coumaric acid (10.15 µg g^−1^), and caffeic acid (1.01 µg g^−1^) [9]. On the other hand, the highest concentration of (−)—epicatechin was found in green tea infusions (83.95 µg g^−1^), almost twice as much as in oolong tea [9]. In turn, fresh leaves of *Camelia sinensis* are richer source of gallic acid (2.37 mg g^−1^) and caffeic acid (92.0 µg g^−1^) compared to black, green, and oolong tea and fermented leaves of *Camelia sinensis* (831.3 and 37.5 µg g^−1^) [4]. Research shows that average intake of total polyphenols with the total diet in Poland is 989.3 mg day^−1^ [10]. The research published in 2018 shows that the daily consumption of polyphenolic compounds along with water infusions prepared from hawthorn fruits (*Crataegus fructus*) and inflorescences (*Crataegus inflorescences*) is approximately 4.7% (46.58 mg day^−1^) and 15.12% (149.56 mg day^−1^), respectively [7]. Knowledge of the content of biologically active compounds in infusions prepared from fresh or fermented plant materials is extremely important from the pharmacological and dietary point of view.

Nowadays, various analytical techniques including high-performance liquid chromatography (HPLC) [4,11,12,13,14], gas chromatography (GC) [15], inductively coupled plasma mass spectrometry (ICP-MS) [16,17], tin layer chromatography (TLC) [13,18], inductively coupled plasma optical emission spectrometry (ICP-OES) [12], atomic absorption spectrometry among (AAS) [19], and inductively coupled plasma-atomic emission spectrometry (ICP-AES) [17] are used for qualitative and quantitative analysis of various bioactive components in “teas”. However, high performance liquid chromatography (HPLC) and gas chromatography (GC) are most commonly used. See Table 1. However, HPLC has several limitations compared to GC or capillary electrophoresis (CE). First of all, the time of analysis and optimization of the method, which often requires a large consumption of solvents and long separation time [5]. An important limitation of GC is that it allows the determination of only those compounds that are volatile and not highly polar, which is of great importance in the analysis of polyphenols, coumarins, organic acids, and vitamins.

High-performance CE is one of the methods used to separate, identify, and quantify various chemical compounds in food products. In recent years, it has become an increasingly popular technique for the separation of analytes. CE is estimated to be the most developing separation technique in the last 20 years. Now CE is increasingly used for powerful separation of different compounds in food and more and more often many researchers use it in the analysis of herbal raw materials. Moreover, according to the World Health Organization, it is recommended to use CE to check the authenticity of herbal raw materials [20]. The availability of high-performance CE enables its wide application in environmental samples [21], pharmaceutical analysis [22], as well as food analysis [23,24].

Due to the higher resolution, CE is more preferred than HPLC in the determination of several components of plant raw materials [25]. Compared to HPLC, GC, or HPTLC, CE is a relatively new analytical technique used for the chemical analysis of multicomponent samples. For the first time in the world, CE was used by A. W. Tiselius in 1937 to separate proteins [26]. After that time, using the PubMed and Web of Science databases, the greatest expansion of works devoted to this technique was noticeable after 1990.

**Scheme 1:**
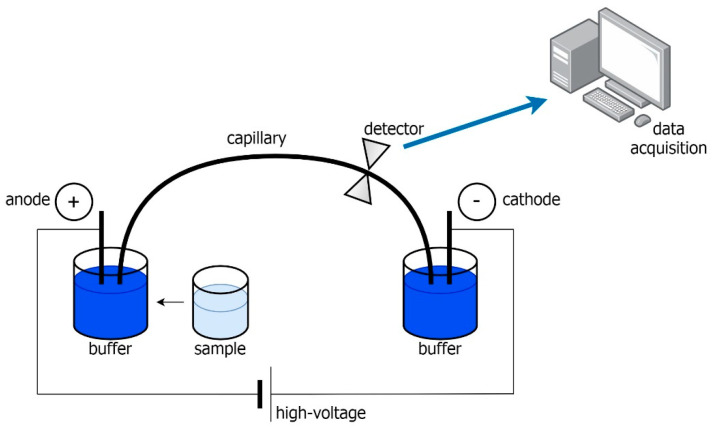
Capillary electrophoresis system.

Whereas in CE, the essence of separation is the electric field. The movement of the mixture components is determined by the electroosmotic flow (EOF) of the buffer and the action of the electric field [27]. The liquid flow in the capillary is due to the potential difference. The speed of an electrically charged particle depends on its charge, size, shape, and resistance. The analyzed compounds, which have an electric charge, are characterized by electric mobility, which causes them to separate. CE resolution is influenced by the total charge in the capillary walls and matrix. Therefore, the pH of the buffer is most important as it dictates the number of theoretical plates. The phenomenon of electrophoresis consists in the migration of charged particles in an electric field towards the electrode with the opposite sign what was shown in Scheme 1. The most important element of the apparatus is the capillary. Their dimensions and type of material (fused silica, or quartz) may affect the analysis conditions. The use of quartz capillaries has the advantage that they can also function as measuring cells of UV/Vis or fluorescent detectors. The flow of electric current through the narrow capillary causes the generation of Joule heat, which is a disadvantageous effect due to the blurring of the component zones of the mixture. Low conductivity, which depends on the material from which the capillary is made, allows the use of a very high electric field (100–500 V cm^−1^), which in turn generates a small amount of Joule heat. In turn, the undeniable advantages of CE include high efficiency, low consumption of reagents, small sample volumes, short analysis times, and the possibility of measuring in a wide range of pH [27,28]. However, an extremely important factor that determines the choice of using CE is that it enables the simultaneous determination of cations and anions [28,29,30]. Due to the use of capillaries with very small diameters, the volume of the introduced sample may reach several nL. For this reason, it is very important in capillary electrophoresis to select an appropriate detection method. The most common are UV/Vis diode array detectors, fluorescence, and amperometric detectors [31]. Unfortunately, the use of small sample volumes can reduce the sensitivity and precision of CE results, what can be avoided by using solid phase extraction (SPE) or optimization of the pre-conditioning and washing step prior to injection [31]. The choice of detector in chemical analysis plays an important role in expressing selectivity. It is obvious that detectors greatly influence the limits of detection (LOD) [32].

An undeniable advantage of CE is the ability to adapt various types of CE depending on the chemical structure of the analyzed compounds. There are several techniques of CE: capillary zone electrophoresis (CZE), non-aqueous CE (NACE), micellar electrokinetic chromatography (MEKC), capillary electrochromatography (CEC), capillary isotachophoresis (CITP), capillary isoelectric focusing (CIEF), chiral CE (CCE), capillary gel electrophoresis (CGE), and microemulsion electrokinetic capillary chromatography (MEEKC) [33].

This article is a valuable summary on the analysis of bioactive compounds in plant materials and their infusions, commonly used “teas”. This issue is important because there is still no data on the amount of consumed substances with water infusions or decoctions. According to data from the last 16 years, CE enables the determination of a wide range of chiral compounds and enantiomers, such as polyphenols, coumarins, alkaloids, vitamins, and ions. This paper discusses in detail the physical (length and diameter of capillary, voltage) and chemical (type of buffer, pH) parameters of CE needed for the selective separation of biologically active compounds. This article summarizes the current application of CE in the analysis of bioactive compounds in plant raw materials, but also indicates the prospects for further applications of this technique.

## 2. Results

### 2.1. Literature Analysis

In the first search in the PubMed and the Web of Science database, 721 records potentially meeting the included criteria were found, 154 and 567, respectively. Then, after reviewing the bibliography, remove duplicates (*n* = 194), and selected articles were subjected to subsequent verification by co-authors. After that, the articles were selected based on the title and abstract. Papers describing the application of different methods than CE or the using CE technique to the analysis of compounds (polyphenols, organic acids, and amino acids) present in spices, vegetables and fruits, blood serum and urine or drugs were eliminated. There were 467 abstracts and papers, but they were not qualified for this review. Fifty-nine articles found in the PubMed and the Web of Science databases were used to review the analysis of various bioactive compounds using CE in *Camelia sinensis* and infusions prepared from dried plant materials commonly known as teas.

### 2.2. Analysis of Plant Material Used in Included Articles

The literature review showed that as much as 62% articles included in this paper comes from China, 20% articles from Europe, 15% from Asia, and only 3% papers from South America. The material covered by the research were parts of plants used as infusions and decoctions belonging to the following families: Onagraceae, Fabaceae, Chloranthaceae, Asteraceae, Hypericaceae, Rubiaceae, Lamiaceae, Compositae, Rosaceae, Pentaphylacaceae, Umbelliferae, Schisandraceae, Rutaceae, Oleaceae, Malvaceae, Nymphaeaceae, Ranunculaceae, Berberidaceae, Ranunculacese, Leguminosae, Gesneriacese, Gentianaceae, Seriphidium, Caprifoliaceae, Phellodendron, Papaveraceae, Nymphaeaceae, Lenguminosae, Apiaceae, and Sapindaceae. Parts of the plants covered by this review are widely used in traditional medicine. CE has been mostly used to analyze bioactive substances in various types of herbs medicine (e.g., *Salvia* species, *Hibiscus sabdariffa, Melissae herba,* or *Scutellaria baicalensis*). In cited works, green tea and tea of unknown species were used six times. Oolong tea, black tea, and jasmine tea, twice; *Camelia sinensis*, once; and rooibos, honey bush, red tea, and mate tea were repeated only three times.

### 2.3. Capillary Zone Electrophoresis

#### 2.3.1. Capillary Zone Electrophoresis with UV Detection

The most widely used method for the selective separation of bioactive compounds in plant raw materials was CE with UV detection (CE-UV). See Table 2. Thanks to UV detectors, it is possible to determine analytes directly or indirectly. The latter method is used when the analyzed substances poorly absorb ultraviolet light [34]. For this, chromophore ions such as phthalic acid, 3,5-dinitrobenzoic acid, or 2,3-pyrazine dicarboxylic acid were added to the buffer for the separation of organic compounds [34]. CE-UV is also used as reference method [35].

CE with UV detection was used for polyphenols compounds analysis in chamomile flowers [36] *Cynanchum chinense* [27], *Salvia* species [37,38,39], *Radix Scutellariae* [20], *Gentiana lutea* [40], mate herb [41], black tea [42], rooibos and honeybush tea [43], flavonoids in *Crataegus monogyna* [44], *Coreopsis tinctoria* [45], and flavone derivatives in *Seriphidium santolinum* [46], fewflower Lysionotus herb [47]. Moreover, this techniques was used to determinate organic acids in sage [48], *Pterocephalus hookeri* [49] and chamomile, linden, and mint [50]. Moreover, CE-UV was used to identify alkaloids in tea [51], *Sophora tonkinensis* [52], *Chelidonium majus* [53], *Cortex Phellodendri Chinensis* [54], *Nelumbo nucifera* [55], anthraquinones in slimming tea [56], coumarins in *Cacalia Tangutica* [57], and nitrate, nitrite, and bromate in tea infusions [30].

To obtain a good separation of the analytes, appropriate conditions of analysis should be selected: such as capillary length, voltage, pH, composition, and buffer concentration. The most appropriate buffer to obtain a good resolution and separation of polyphenolic compounds was borate buffer.

To begin with, 40 mM L^−1^ borate buffer was used to separate of (−)-epicatechin, catechin, vanillic acid, rosmarinic acid, caffeic acid, and gallic acid from *Salvia* species [38]. A 50 and 100 mM L^−1^ sodium tetraborate solution was used in isolation of apigenin-7-glucoside and apigenin from chamomile [36]. Rosmarinic acid (RA) and carnosic acid (CA) are antioxidant compounds in *Salvia* species and were determined with use of a background electrolyte (BGE), which constituted of 20 mM L^−1^ borate at 9.6 pH. The average amount of real samples ranged within 1.08–14.40 mg g^−1^ DW for RA and 11.0 mg g^−1^ for CA [37]. This method was validated and LOD for RA was 1.72 µg mL^−1^ and 1.86 µg mL^−1^ for CA. CE-UV (254 nm) was used for determination of two flavonoids 7-*O*-α-L-rhamnopyranosyl-kaempferol-3-*O*-β-D-glucopyranoside (GL) and 7-*O*-α-L-rhamnopyranosyl-kaempferol-3-*O*-α-L-rhamnopyranoside (RH) [27]. Authors determined GL and RH in *Cynanchum chinense* using 30 mM L^−1^ borate at 9.50 pH. Under this condition authors obtained LOD 2.1 and 1.6 µg mL^−1^ for GL and RH but concentration of this two flavonoids were in the range 0.151–0.284 mg g^−1^ for GL and 0.502–20.412 for RH [27].

In chiral separation, the addition of β-cyclodextrin (β-CD) to the buffer is often used to prove the distribution of the analyzed ions. This kind of modification was used in determination of seven phenolic acids in *Salvia* species [39] in within 17 min, oleanolic and ursolic acid in *Pterocephalus hookeri* [49] in less than 11 min, gentisin, isogentisin, and amarogentin in *Gentiana lutea* [40] and alkaloids in *Sophora tonkinensis* [52]. Cao et al. determined the highest concentration of RA in *Salvia castanea* from Yunnan (21.42 ± 0.73 mg g^−1^) but the lowest in *Salvia miltiorrhiza* from Anhui (1.30 ± 0.33 mg g^−1^) [39]. In turn, *Salvia miltiorrhiza* (Hebei) was the richest (44.61 ± 0.72 mg g^−1^) in salvianolic acid B but *Salvia castanea* from Yunnan was only 1.66 ± 0.07 mg g^−1^ this acid [39]. Application of CE-UV allowed on determination gentisin in *Gentiana lutea* in the range of 4.27–9.72 µg mL^−1^ but amarogentin was not detected in two samples. In turn, the highest concentration of isogentisin (12.30 µg mL^−1^) was found in the sample without amarogentin [40].

The efficient separation of phenolic compounds such as rutin (R), caffeic acid (CAA) and 3,4-dihydroxybenzoic acid (3,4-DHBA) in mate herb with the 100 mM L^−1^ boric acid (pH 9.0) allowed the analysis time to be reduced to 4 min [41]. Moreover, authors obtained low limit of detection of 0.14, 0.05, and 0.05 mg L^−1^ for R, CAA and 3,4-DHBA, respectively. The content of R and CAA before hydrolysis was in the range of 343.55–516.74 and 3.77–7.58 mg 100 g^−1^, respectively, but 3,4-DHBA was not identified. Additionally, no R was found after the hydrolysis process. In turn, concentration of CAA and 3,4-DHBA in mate herb was in the range 443.14–737.49 and 24.85–35,59 mg 100 g^−1^, respectively [41].

Three buffers with different range pH were used to optimize the separation of epicatechin, epigallocatechin gallate, morin, chrysin, and hesperidin: sodium tetraborate (pH 8.0–10.0), sodium hydrogen phosphate (pH 7.5–9.0), and sodium acetate (pH 6.0–7.0) [42]. Therefore, sodium tetraborate buffer with 1-butyl-3-methyl imidazolium hexafluorophosphate (BMIM-PF_6_) as an additive was chosen for analysis [42]. Under this separation conditions, five different compounds could be completely separated in 8 min. Authors showed that average concentration of epigallocatechin gallate (EGCG) and epicatechin (EC) in green tea samples was higher compared to black tea. Average content of EGCG and EC was 28.36 and 15.60 mg g^−1^ for green tea and 14.02 and 6.65 mg g^−1^ for black tea [42]. In the other studies, selected polyphenols compounds were isolated in tea rooibos (*Aspalathus linearis*), and honeybush (*Cyclopia substernata* and *Cyclopia maculata*) [43]. In this research, optimal separation for honeybush and rooibos phenolics was achieved in 21 and 32 min. In pursuit of the most optimal separation conditions, Urbonavičiute et al. originally conducted studies using buffer 50 mM L^−1^ Na_2_B_4_O_7_ at pH 9. However, at pH 8.2, the separation of rutin, vitexin-2″-*O*-rhamnoside and hyperoside was the best [44]. In quantitative analysis of polyphenols in *Coreopsis tinctoria* authors used running buffer at different pH values, between 8.0 and 10.0. The best selectivity obtained with pH 9.0 borate buffer [45]. The contents of flavonoids and phenolic acids were decreased in the following: flower > bud > seed > leaf and >stem. Authors observed that okanin 4′-*O*-glucoside was the most abundant in dried flower—33.8–60.9 mg g^−1^ [45]. In addition, the optimal BGE composition for the selective separation of arteanoflavone, eupatilin, hispidulin, and 5, 7,4′-trihydroxy-6,3′,5′- trimethoxyflavone in Chinese herbs was 25 mM L^−1^ borate and 6 mM L^−1^ β-cyclodextrin (β-CD) [46]. In turn, using of 30 mM L^−1^ borax solution (pH 10.2) allowed an identification of nevadensin in Fewflower Lysionotus Herb on the level of 2.82 mg g^−1^ (RSD 3.22%) [47]. Application of 40 mM L^−1^ borate buffer and shorter fused silica capillary (53 cm) in comparison to the previously cited Adimicilar et al. studies, higher detection limits for carnosic acid (CA) and rosmarinic acid (RA) in sage were 2.79 and 3.18 µg mL^−1^, respectively [48]. Studies have shown a higher concentration of CA (5.26 mg g^−1^) compared to RA (3.14 mg g^−1^).

For the purpose of quantifying of succinic (SA), malic (MA), tartaric (TA), citric (CITA), and lactic acid (LA) in chamomile, linden, and mint, a 0.5 M H_3_PO_4_ and 0.5 mM L^−1^ cetyltrimethylammonium bromide (pH 6.25) was used [50]. Application of indirect UV detection caused that average content of SA in decoction from chamomile was in the level of 9.98 mg L^−1^ but in infusion was not detected. In turn, MA was identified in all analyzed samples in wide range of 18.20–111.53 mg L^−1^ and TA was only in infusion and decoction mint (19.02 and 24.76 mg L^−1^) [50]. The separation of alkaloids in tea was obtained using 9.2 pH 15 mM L^−1^ borax (BGE) [51]. Under this condition, the LOD was 3.0 for caffeine, 2.1 for theobromine, and 1.6 µg mL^−1^ for theophylline and the concentration of these analytes in the real samples were 34.30, 2.87, and 2.64 mg mL^−1^, respectively. Zhou et al. developed for the quantification of eight isoquinoline alkaloids in *Chelidonium majus* [53]. Low pH value of Tris-H_3_PO_4_ buffer (pH 2.5) containing 50% methanol and 2 mM HP-β-CD allowed identification of sanguinarine, coptisine, chelerythrine, berberine, chelidonine, protopine, allocryptopine, and stylopine with low limits of detection in 9 min. See Table 2.

#### 2.3.2. Capillary Zone Electrophoresis with Electrochemical Detection

In recent years, the use of CE with electrochemical detection for the separation and subsequent determination of polyphenols, flavonoids oolong tea, and Chinese herbal tea [25,59] and alkaloids in *Plumula Nelumbinis* [60]. See Table 3.

Researchers have high hopes for application of amperometric detection (AD) with CE, because of high sensitivity and selectivity and miniaturization of the detection system [60]. In the case of CE-AD, the miniaturization process consists in the use of reagent volumes from several dozen to several hundred µL [60]. For the determination of alkaloids, Wan et al. used only 100 µL of sample and 1 mL of the running buffer in the study [60]. In the cited article, the alkaloids were separated from *Plumula Nelumbinis* within 12 min in a 40 cm long fused silica capillary with a 50 mM L^−1^ borate buffer (pH 9.2). In this study, the authors used a three-electrode detection cell with a carbon detection electrode with a diameter of 300 µm, a platinum auxiliary electrode and a saturated calomel electrode as a reference electrode [60]. Authors determined neferine, liensinine, isoliensinine, rutin, and hyperoside in the range of 2.24–3.67, 5.14–8.99, 1.11–2.84, 1.35–3.49, and 0.14–0.29 mg g^−1^. Amperometric detection with Cu disc electrode with diameter of 300 µm as working electrode, saturated calomel reference electrode and a Pt electrode was used for detection of L-theanine (L-THE), L-glutamine (L-GLU), sucrose (SUC), glucose (GLU), fructose (FRU), ascorbic acid (ASC), and (−)-epigallocatechin gallate (EGCG) [25]. The authors argue that due to complexation, amino acids, such as L-THE, L-GLU may respond well to the Cu disc electrode. Thanks to the application this kind of detector concentration of L-THE, L-GLU, SUC, GLU, FRU, and EGCG in oolong tea originated from China were 0.03, 0.03, 0.72, 0.14, 0.33, and 0.10 mM L^−1^. In turn, ASC was not detected in analyzed samples [25]. Due to the similar structure of the antioxidant compounds, the separation of kaempferol (K), apigenin (A), rutin (R), ferulic acid (FA), quercetin (Q) and luteolin (LUT) is difficult. This problem was solved by adding 0.20 mM L^−1^ β-CD to the buffer [59]. Under the optimum CZE-AD conditions, it was found of K, A, R, FA, Q, and LUT in the average level of 0.74, 0.36, 0.61, 0.53, 0.35, and 0.55 µg 100 mL^−1^, respectively [59].

Three electrodes, 500 µm diameter carbon disc working electrode, a Pt auxiliary electrode and a Ag/AgCl reference electrode in combination with an amperometric detector was used for determination of catechin (CAT), rutin (R), hyperoside (H), quercetin (Q) and quercitrin (QU) in *Agirimonia pilosa* [61]. Under these conditions, it was found that the *Agrimonia pilosa* stems were a richer source of R and H and poorer of CAT, Q and QU. The highest concentration of H was determined in stems (576.0 µg g^−1^) [61]. The use of an ultra-small sample volume, the consumption of low solvent volumes and a simple pre-treatment of the sample were proposed in studies with the herb *Acanthopanax senticosus* [62]. The best resolution and the higher peak currents for isofraxadin (ISOF) and rutin (R) could be possible with use of mixture 7.5 mM NaH_2_PO_4_ and 7.5 mM borax (pH 6.0). The lowest concentration of ISOF was found in leaf (1.2 µ g^−1^) and root (1.2 µ g^−1^) of *A. senticosus* and R was isolated only in leaf (13.0 µ g^−1^). Moreover, in the Zhou et al. research 33 µm carbon fiber microdisk electrode (CFE) has been applied to identification of aristolochic acid I (AA-I) and aristolochic acid II (AA-II) [63]. In this paper, the optimum condition to separation of this two compounds was 20 mM L^−1^ phosphate buffer solution with pH 10.0. Modification of CE-ED technique allowed for obtained low LOD, which is equal for AA-I and AA-II 0.04 and 0.01 µM, respectively. The concentration of AA-I in the root of *Aristolochia debilis* was more than twice higher than in AA-II [63].

#### 2.3.3. Microfluidic Analysis with Contactless Conductivity Detection

In recent years, more and more attention has been paid to new, more improved solutions with the use of lab-on-a-chip microfluidic devices [65]. They enable fast and highly sensitive analysis and improvement of the repeatability of analyzes. The miniaturization of analytical processes has brought great hope in recent years. This is due not only to the small volumes of the reagents, but also to the reduced analysis time and low energy consumption [66].

Microfluidic analytical system (MFAS) was used for determination of epigallocatechin gallate (EGCG), epicatechin (EC), epicatechin gallate (ECG), and epigallocatechin (EGC) in green tea [65]. In this paper, authors manufacturing a chip based on polydimethylsiloxane and glass. Under optimum conditions (20 mM borate buffer electrolyte, pH 9.2) LOD for EC, EGC, ECG, and EGCG was 3.5, 3.5, 3.2, and 2.3 µg mL^−1^. The concentration of EGCG, GCG, EC, ECG, and EGC was 167 ± 15, 44 ± 8, 162 ± 15, 67± 9, and 4 ± 2, respectively [65]. Application of conductively detector is very sensitive and suitable. Tang et al. stabilized the ionic strength by adding lactic acid and β-alanine as background electrolyte components in the CE method with capacitively coupled contactless conductivity detection (CE-C^4^D) [67]. C^4^D is suggested when the molecules are without or weak chromophores and when their detection with optical systems is impossible. This procedure enabled the determination of eight metal ions (Mg^2+^, Mn^2+^, Cd^2+^, Co^2+^, Pb^2+^, Ni^2+^, Zn^2+^, and Cu^2+^) in dried *Forsythiae Fructus* (Oleaceae) in 10 min, which possesses anti-inflammatory, antioxidant as well as hepatoprotective, neuroprotective and cardiovascular protective effects [67]. The highest concentration of analyzed compounds was found for magnesium (2.38–4.14 mg g^−1^) what is important from the nutritional point of view [68].

In recent years, unique solutions have also been sought, such as the use of home microchip electrophoresis with an integrated Pt detector [66]. This type of solution was used to analyze guanosine (G), methionine (M), glycine (GLY), 3,4-dihydroxybenzaldehyde (DHB), and homogentisic acid (HA) in *Pinellia ternata* used in traditional Chinese medicine. Using an innovative solution, Shih et al. determined five ingredients were determined within 5 min using the special platform [66]. Using this techniques authors determined G, M, GL, DHB, and HA on the average level of 141.5, 20.5, 25.1, 44.3, and 62.6 µg g^−1^. Strychalski et al. developed simple and robust analytical technique, well-suited to microfluidic, called GEMBE (gradient elution moving boundary electrophoresis) [69]. This technique uses ultrasmall capillary or microchannel (few mm–several cm in length). In this study 5.5 cm long fused silica capillary (OD 363.5 and ID 13.5 µm) was used [69]. Detection point was only approximately 15 mm from the capillary inlet. The essence of this method is also the use of much lower volumes of solutions (200 µL) [69]. In a complex matrix such as tea, CE chips with electrochemical detection are selective micro-flow microfluidic platforms [70]. Two analytical solutions were tested in these studies: class-selective electrochemical index determination (CSEID) and individual antioxidant determination (IAD). For CSEID optimal results were obtained for the separation of flavonoids and phenolic acids in less than 100 s using MES (2-(N-morpholino)ethanesulfonic acid) at pH 5 and for IAD separation of nine phenolic compounds was provided in a borate buffer at pH 9 in only 260 s. In this paper, authors determined (+)-catechin, rutin, ferulic acid, chlorogenic acid, vanillic acid, quercetin, caffeic acid, gallic acid, and protocatechuic acid in the total level of 290 ± 2 for CSEID and 321 ± 11 µg mL^−1^ in green tea [70].

#### 2.3.4. Capillary Zone Electrophoresis with Fluorescence Detection

The literature review shows that in CE-UV detection is also replaced by fluorescence detection (Table 4). Combination of CE with laser induced fluorescence (LIF) has provided improvement in detection limit, compared with UV detector [71]. This type of detection was used to determine the amino acids in tea infusions, oolong tea and jasmine tea [72,73,74] and riboflavin in *Camelia sinensis* [71] and green tea name Zhuyeqing [75]. CE derivatization methods were used to determine γ-aminobutyric acid (GABA) and alanine in aqueous extract of Chinese tea after derivatization with *o*-phtaldialdehyde/2-mercaptoethanol (OPA/2-ME) to produce fluorescently labeled analytes [72]. The labeled derivatization with 20 mM L^−1^ OPA and 26.67 mM L^−1^ 2-ME at pH 10.0 gave the most sensitive detection and optimum buffer was composed with 30 mM L^−1^ sodium tetraborate (pH 10.0) for determination of these two amino acids in tea. Under this conditions authors obtained LOD 0.004 and 0.02 µM for GABA and alanine. In turn, GABA was determined in jasmine green tea, oolong tea and GABA-rich tea on the level of 2.5, 6.0, and 157.2 mg 100 g^−1^, respectively, and alanine was identified at 22.7, 13.3, and 51.8 mg 100 g^−1^, respectively [73]. Moreover, amino acids was analyzed with use of combination of CE and light-emitting diode-induced fluorescence detection (LED-IF) [74]. Authors claimed that by using 0.5% PEO solution (prepared in 10 mM L^−1^ Na_2_B_4_O_7_ at 9.3 pH) and 60 cm capillary length, GABA, GL, and aspartic acid (ASP) were marketed within 16 min. Moreover, fluorescence detector was used in the analysis of riboflavin (RF) concentration in *Camelia sinensis* [71,75]. RF was determined in green tea by CE with in-column optical fiber laser-induced fluorescence detection (CE-LIF). The concentration of RF in samples were between 0.05 and 20 µM with LOD 3.0 nM [75].

In the other studies, application of non-aqueous capillary electrophoresis (NACE) was coupled with laser-induced native fluorescence detection for analysis of three alkaloids in methanol extract prepared from dried *Rhizoma coptidis* and *Caulis mahoniae* [76]. The authors consistently changed the analysis conditions to obtain the shortest possible analysis time and to generate the lowest Joule heat possible not to cause overlapping of the mixture component zones. In the course of the conducted experiments, it was noted that with the increasing percentage of ammonium acetate in BGE, the migration time increased. The strongest detector signal was obtained with 35 mM L^−1^ ammonium acetate. That is way, the authors obtained the optimal conditions using 5% acetonitrile, 0.25% acetic acid and 35 mM L^−1^ ammonium acetate in methanol [76]. The use of fluorescence detector allowed to obtain a lower LOD compared to UV detection. For palmitine (PAL), authors received LOD on the level of 7.5, for berberine (BER) 6.0 and jatrorrhizine (JATR) 380.0 ng mL^−1^. Under this conditions, BER and PAL was identification on the average level of 4.95 ± 0.23 and 2.92 ± 0.17% for *R. coptidis* and 1.02.± 0.08 and 0.59 ± 0.03% for *C. mahoniae* [76]. Previously, the aqueous electrolyte was not suitable for the MS system, therefore, non-aqueous CE with UV and MS detection was used to determine the alkaloids in *Nelumbo nucifaera* [55]. In turn, using the fluorescence detection has its limitations as not all compounds are capable of fluorescence, which is necessary for the application of this type of detector in quantification. Only Wang et al. determined naringin, esculin, genistein, isofraxidin, and esculetin in Fructus Sophorae japonicae and Herb sarcandrae belonging to the Chloranthaceae family [77].

### 2.4. Micellar Electrokinetic Chromatography

Another kind of CE is micellar electrokinetic chromatography (MEKC) used mainly for the separation of mixture components whose analytes are electrically inert and charged. An important difference that distinguishes this type of CE is the use of a surfactant in the buffer to form micelles. The result is a pseudostationary phase (micellar phase) and a mobile liquid phase.

This technique was used for the analysis of polyphenols in *Scutellaria baicalensis*, tea samples, oolong tea, and green tea, *Salvia officinallis* [78], tea samples [79], and *Arnica montana* [80]. In turn, catechins and methylxanthines in green tea samples and coumarins in *Aesculus hippocastanum* and *Heracleum sphondyliu* [81]. Moreover, amino acids in black, jasmine, green tee [82], and indoleamines (melatonine) in *Camelia sinensis* and *Tilia cordata* [83]. The full characteristics of the method used are presented in the Table 5. The most common surfactant used in the MEKC technique is SDS (sodium dodecyl sulfate). Buffer system containing 15 mM L^−1^ borate, 40 mM L^−1^ phosphate and 15 mM L^−1^ SDS with 15% acetonitrile and 7.5% 2-propanol was used to separate baicalin (B), baicalein (BC) and wogonin (W) in *Scutellaria baicalensis* originated from China, where baseline separation was obtained within 15 min [78]. In this research, in analyzed samples concentration of B, BC and W were determined in the range of 24.74–143.56 mg g^−1^, 1.53–15.12 mg g^−1^, and 0.37–4.80 mg g^−1^. Similarly, SDS has also been used to separate catechins from green tea [79,84]. But, for the separation of six major green tea catechins and enantiomers of theanine was used Heptakis (2,6-di-*O*-methyl)-β-cyclodextrin [84] and hydroxypropyl-β-cyclodextrin (HP-β-CD) as chiral selector [79]. Moreover, Gomez et al. used a 10 mM L^−1^ sodium tetraborate (pH 9.2) as a BGE and mixture of 20 mM L^−1^ SDS anionic micelles and 20 mM L^−1^ β-CD with 10% acetonitrile in identification of antioxidative melatonin [85] in *Camelia sinensis* and *Tilia cordata*. This allowed the detection to be reduced to low ppb levels [83]. The average content of melatonin in green tea was 386 ng g^−1^ [83]. To isolate the coumarins (isopimpineline, bergapten, phellopterin, esculin, and esculetin) from *A. hippocastanum* and *H. sphondylium* originated from Poland, Dresler et al. replaced SDS with 65 mM L^−1^ SC (sodium salt) [81]. Using the 50 mM L^−1^ sodium tetraborate and 60 mM L^−1^ SC and 20% methanol (*v*/*v*) authors ensured good resolution of the analyzed compounds. Similarly, during the analysis of amino acids in aqueous infusions of tea, 20 mM L^−1^ sodium borate (pH 8.5) and 20 mM L^−1^ Brij 35 with 10% acetonitrile was used [82]. Due to the long analysis time and the poor separation efficiency, popular SDS was changed to Brij 35. Under this separation conditions, 15 different amino acids could be completely separated in 11 min.

### 2.5. Capillary Isotachophoresis

Capillary isotachophoresis (CITP) is an anion-cation separation technique in which, unlike CZE, two buffer systems are used. One of them is called the leading electrolyte (LE) with high ion mobility compared to the analyte and terminating electrolyte (TE) with reduced ion mobility. This kind of technique of CE was used in two articles to analyze selected antioxidants in *Melissae herba* and in *Herba Epilobi* which are traditionally used for the symptomatic treatment of gastrointestinal disturbances and for urological problem in men, respectively [87,88,89], which was shown in Table 6. Using 10 mM L^−1^ HCl and 0.2% hydroxyethylcellulose as LE buffer and 50 mM L^−1^ H_3_BO_3_ as TE buffer, CA, RA, *p*-coumaric acid (*p*A), chlorogenic acid (CLA), FA and QU were determined in *Melissa herb* at the level 1.65 ± 0.80, 43.54 ± 1.73, 1.00 ± 1.04, 0.30 ± 4.65, 3.70 ± 2.17, and 1.25 ± 2.97 mg g^−1^, respectively [88]. Using of CITP method authors obtained limit of detection at level of 0.018 for CA, 0.027 for RA, 0.030 µg mL^−1^ for the *p*A, 0.032 for CLA, 0.020 for FA and 0.035 µg mL^−1^ for QU. In turn, the combination of capillary isotachophoresis (ITP) and capillary zone electrophoresis (CZE) was used to determinate phenolic acids in Herba Epilobi, which could allow to obtain LOD on the level 0.05 for cinnamic acid, 0.010 for *pA*, 0.021 for FA, 0.026 for syringic acid, 0.034 for CA, 0.041 for protocatechuic acid, 0.044 for vanillic acid, and 0.061 µg mL^−1^ for CLA [87]. 

### 2.6. Capillary Electrochromatography

Capillary electrochromatography (CEC) is an electrokinetic separation technique [90]. Using the CEC technique it is possible to separate uncharged and charged substances. CEC combines elements of two techniques: capillary electrophoresis (CZE) and high performance liquid chromatography (HPLC). In CEC it is possible to use packed, monolithic, and open-tubular columns (OTC). However, in recent years, monolithic and open-tubular columns have been used more frequently [90]. OTC allows the use of innovative microporous materials, nanoparticles, and biomaterials as stationary phase elements, which gives wide analytical possibilities. In turn, monolithic columns have higher efficiency and resolution compared to OTC [90]. CEC combines the advantages of both techniques, HPLC and CE. On the one hand, the retention of the analytes depends on their interaction with the surface of the stationary phase particles. On the other hand, in the case of electrically charged elements, it also depends on their electrophoretic mobility. This, in turn, is strongly influenced by the strength of the electric field, the composition of the mobile phase, the ionic strength and the pH of the buffer [91].

The methanolic extracts of *Adinandra nitida* was analyzed with use of monolithic columns of CEC [92]. Using 10 mM L^−1^ ammonium formate (3.0) as BGE, separation of flavonoids, e.g., EC and A, could be accomplished in 25 min on a monolithic rod of macroporous poly(butyl methacrylate-*co*-ethylene dimethacrylate). In turn, during the analysis of 11 coumarins, flavones, and flavanone (Table 7) in *Chamomilla recutita*, the Hypersil SCX/C18 column with phosphate buffer (pH 2.8) at 50 mM L^−1^ with 50% acetonitrile was used [93]. These conditioned parameters could separate all compounds in less than 7.5 min under isocratic conditions, and moreover, the LOD for A with UV detection at 337 nm was 35.0 µg mL^−1^. In other studies, good resolution of (+)—catechins, (−)—epicatechins, (−)—epigallocatechins, theophylline, and caffeine in black and green teas were used with a capillary column (ID 100 µm) filled with C18 bidentate particles at 24.5 cm. The mobile phase was a mixture of 5 mM L^−1^ ammonium acetate buffer (pH 4.0) with H_2_O/acetonitrile (80:20, *v/v*) and LOD for all analyzed compounds with UV detection at 200 nm was 1.0 µg mL^−1^ [94]. Moreover, capillary electrochromatography was used to determinate six coumarins in *Fructus cnidii* ethanolic extracts [95]. The separation efficiency of the methods was performed in an in-house packed column with a monolithic outlet frit with 10 mM L^−1^ ammonium acetate buffer (pH 4.0) and 50% acetonitrile in 15 min. Limit of detection for bergapten, imperatorin, osthole, 2′-acetylangelicin, oroselone, and *O*-acetylcoumbianetin was 2.5, 5.0, 1.0, 2.5, 2.5, and 2.5 µg mL^−1^, respectively. Another type of detection was used in Liu et al. research, where authors determined evadiamine, rutaecarpine, and limonin in *Evidiae fructus* fruit [5]. As stationary phases was used home-developed monolithic columns with methyl-vinylimidazole functionalized organic polymer monolilth. In this study CEC-MS and CEC-UV were compared. Authors obtained LODs of three analyzed compounds in the range of 2.0–12.5 µg mL^−1^ by UV detector and 0.12–3.1 µg mL^−1^ by MS detector. Studies have confirmed that the use of CE with MS detection increases the sensitivity of the method several times, which allows for the determination of alkaloids and limonoids in plant materials [5].

### 2.7. Capillary Electrophoresis-Mass Spectrometry

CE can be coupled with mass spectrometry detector (CE-MS) what was shown in Table 8 [96,97]. The essence of CE-MS is the electrokinetic separation of analyte groups as a result of the mobility of ions in the electric field [97]. Thanks to the use of MS, it is possible to explain the molecular structure of metabolites that cannot be obtained by other detection methods, e.g., UV detector [5]. MS detection distinguishes the target analyte signal from the sample of the composite matrix and eliminates high background noise. The use of an MS detector may result in a higher sensitivity and selectivity of the analysis compared to the UV detector [5].

Studies with use of four alkaloids standards (coptisine, berberine, palmatine, and jatrorhizine) found in *Rhizoma coptidis* proved that 1000 times lower LOD with the use of CE-MS compared to UHPLC-MS [96]. SPE-CE-ESI-MS was used to develop a method of separation and identification of anthocyanins in *Hibiscus sabdariffa* showing antihypertensive and cardioprotective effects [97]. Using 200 mM L^−1^ boric acid and ammonia (9.0) as BGE, separation of chlorogenic acid, delphinidin-3-*O*-glucoside, cyaniding-3-*O*-rutinoside, cyaniding-3-*O*-sambubioside, and delphinidin-3-sambubioside in *Hibiscus sabdariffa* could be accomplished in below 20 min [97].

## 3. Materials and Methods

This review is based on PRISMA guidelines. The articles selection criteria for the review were carried out using PICOS (Population, Intervention, Comparison, Outcome, Study type) process. For the purpose of this review, articles from 2005 to 2021 were used. Searching of literature for this publication was performed between November 2020 and January 2021 using the PubMed and Web of Science database. The search strategy was with use of the following keywords:

1. “capillary electrophoresis” and “raw material”,
2. “capillary electrophoresis” and “tea”, and
3. “capillary electrophoresis” and “herb”.

In the PubMed base a combination of terms “All fields” and in Web of Science base terms “Topic” was used, which searches titles, abstracts, author keywords, keywords Plus. Only articles in English, available full texts and articles containing publications focused on the analysis of bioactive compounds in plant raw materials by CE are included in this review. See Table 9. Moreover, the search was limited to the matrix, which was a plant materials commonly used as aqueous infusions (tea) or decoctions in traditional medicine. The exclusion criteria were opinion letters, conferences abstracts, papers not written in English (for examples Chinese). Publications in which ornamental horticulture flowers, vegetables, and spices were used as plant material were rejected. Additionally, articles with urine and blood serum, tablets and capsules as the matrix have been eliminated. Studies in which mycotoxins were analyzed using CE were also not taken into account. Duplicates were removed and next, found articles were sorted by title, abstract and then main text. The articles were excluded if they does not meet the inclusion criteria. Selection of appropriate works taking into account inclusion and exclusion criteria were controlled by three authors of this paper (A. P., M. G., and M. K.). Selection of the publications by them was based on the qualitative and quantitative evaluation of articles from the PubMed and Web of Science database, especially title of paper, first name of author and year publication.

## 4. Conclusions

In this review, the authors summarized the last sixteen years of scientific research using capillary electrophoresis to identify and quantify bioactive compounds in raw materials commonly used as “tea” in China, Europe, Asia, and South America. By far China is an area in the world where the number of scientific reports about analysis of polyphenols, coumarins, alkaloids, or amino acids in dried herbal raw materials is greater than in the rest of the world.

CE’s versatility is primarily due to its many techniques. Among all the capillary electrophoresis methods, the most popular CE is capillary zone electrophoresis with UV detection. With use of this technique, it is possible to analyze a numerous bioactive compounds in dried raw materials in less than 20 min and low limit of detection. Nevertheless, the use of CE-MS allows for the more sensitive determination of elements with a low limit of detection and gives hope for routine use in the analysis of functional foods. Unfortunately, a major limitation in using the MS detector in conjunction with CE may be its incompatibility with some types of CE. Chiral separations using CE-MS are also limited due to the incompatibility of the chiral selectors with the MS detector. Research aimed at developing chiral selectors compatible with MS seems to be the direction of future research by scientists. Furthermore, one of the limitation in the use of capillary electrophoresis is the choice of a chiral selector during optimizing enantiomeric separation. The type and concentration of cyclodextrins, which are used most often, is one of the most important parameters for proper separation. Moreover, the use of some modifications in electrochemical techniques allows to reduce the sensitivity of the methods along with the reduction of the analysis time.

## Figures and Tables

**Table 1:** Number of papers published in the Web of Science database using various analytical techniques.

Web of Science								
Keyword	HPLC	GC	CE	ICP-MS	TLC	ICP-OES	AAS	ICP-AES
“tea”	2912	1665	356	260	149	145	142	134
	(51%)	(29%)	(6%)	(5%)	(3%)	(2%)	(2%)	(2%)

HPLC—High-Performance Liquid Chromatography, GC—Gas Chromatography, CE—capillary electrophoresis, ICP-MS—Inductively Coupled Plasma Mass Spectrometry, TLC—Tin Layer Chromatography, ICP-OES—Inductively Coupled Plasma Optical Emission Spectrometry, AAS—Atomic Absorption Spectrometry, ICP-AES—Inductively Coupled Plasma-Atomic Emission Spectrometry.

**Table 2:** Application of capillary electrophoresis with ultraviolet detection (CZE-UV).

Ref	Instrumental Variables					Chemical Variables		Compounds	λ (nm)	LOD (µg mL^−1^)
	V (kV)	L_cap_ ^ (cm)	ID (µm)	OD (µm)	T (°C)	BGE	pH			
[30]	−20	61.5 FSC	50	nd	nd	50 mM L^−1^ phosphate buffer	3.5	nitrate, nitrite, bromate	200	0.60 0.99 2.14
[35]	25	51.5 FSC	nd	nd	nd	50 mM L^−1^ sodium tetraborate	9.3	vitexin derivative, quercetin derivatives, chlorogenic acid, caffeic acid, isorhamnetin derivative, rosmarinic acid, protocatechuin acid, salvianolic acid, derivative, luteolin derivatives, narigeninin derivatives, dicaffeoylqunic acid derivatives, tannins derivatives	200–400	*
[36]	20	75 QC	50	nd	RT	50 and 100 mM L^−1^ sodium tetraborate	nd	apigenin-7-glucoside, apigenin	254	nd
[27]	20.0	50 FSC	75	nd	RT	30 mM L^−1^ borate buffer	9.50	GL, RH	254	2.1 1.6
[37]	28	67 FSC	50	nd	25	20 mM L^−1^ borate buffer	9.6	carnosic acid, rosmarinic acid	210	1.7 1.9
[38]	20.0	43 FSC	75	nd	25	40 mM L^−1^ borate buffer	9.2	(−)-epicatechin, catechin, vanillic acid, rosmarinic acid, caffeic acid, gallic acid	280	0.2 0.2 0.5 0.8 0.7 1.7
[39]	20	70 FSC	75	375	nd	20 mM L^−1^ sodium tetraborate with 12 mM L^−1^ β-CD	9.0	protocatechuic acid, salvianolic acid, rosmarinic acid, salvianolic acid A, danshemsu, salvianolic acid B, protocatechuic acid	280	0.18 0.14 0.24 0.28 0.26 0.36 0.18
[20]	12.0	75 FSC	75	nd	RT	50 mM L^−1^ sodium borate, 5% acetonitrile, 1 M NaOH	9.3	baicalin	280	nd
[40]	25	60 FSC	50	nd	30	100 mM L^−1^ sodium tetraborate, 10 mM L^−1^ β-CD	9.3	gentisin, isogentisin, amarogentin	260 260 242	0.69 1.22 1.24
[41]	30	40 nd	50	nd	25	100 mM L^−1^ boric acid	9.0	rutin, caffeic acid, 3,4-dihydroxybenzoic acid	217	0.14 0.05 0.05
[42]	17	57 FSC	75	375	25	25 mM L^−1^ borate buffer	9.0	hesperidin, chrysin, epicatechin, epigallocatechin gallate, morin,	214	0.44 0.50 0.48 0.54 0.47
[43]	30	88.5 FSC	nd	nd	20	200 mM L^−1^ borate buffer	8.8 and 9.25	phenylpyruvic acid-2-*O*-glucoside, luteolin-7-*O*-glucoside, isovitexin, isoorientin, vitexin, orientin, chrysoeriol, luteolin, scolymoside, hesperidin, eriocitrin, quercetin, isoquercitrin, hyperoside, rutin, nothofagin, aspalathin, phloretin-3′,5′-di-C-glucoside, hydroxycinnamic acid, iriflophnone-3-C-glucoside- 4-*O*-glucoside, iriflphnone-3-C-glucoside, mangiferin, isomangiferin	283 330 384	0.73–14.9
[44]	25	71 FSC	50	nd	25	50 mM L^−1^ Na_2_B_4_O_7_	8.2	vitexin, rutin, vitexin-2′’-*O*-rhamnoside, hyperoside	280	0.43 0.88 0.53 0.43
[45]	25	50 FSC	75	365	25	50 mM L^−1^ borax, 15% (*v*/*v*) acetonitrile	9.0	tiaxifolin-7-*O*-glucoside, flavanomarein, quecetagetin-7-*O*-glucoside, okanin 4′-*O*-glucoside, chlorogenic acid, okanin	280	3.23 0.95 1.46 2.21 0.58 0.63
[46]	20.0	50 FSC	75	nd	20	25 mM L^−1^ borate, 6 mM L^−1^ β-CD	10.2	arteanoflavone, eupatilin, hispidulin, TTMF	254	0.945 0.762 1.002 1.036
[47]	16	52 nd	75	nd	27	30 mM L^−1^ borax solution	nd	nevadensin	335	nd
[48]	28	53 FSC	50	nd	nd	40 mM L^−1^ borate buffer	9.6	carnosic acid, rosmarinic acid	210	2.79 3.18
[49]	20	62.5 FSC	75	nd	25	50 mM L^−1^ borax and 8 mM L^−1^ β-CD	9.53	ursolic acid, oleanolic acid	214	3.8 3.4
[50]	−10	72 FSC	50	nd	25	50 mM H_3_PO_4_ and 0.5 mM L^−1^ cetyltrimethylammonium bromide	6.25	succinic acid, malic acid, tartaric acid, citric acid, lactic acid	200	0.97 3.10 2.18 2.53 89.73
[51]	20	72 FSC	75	375	nd	15 mM L^−1^ borax	9.2	caffeine, theobromine, theophylline	274	3.0 2.1 1.6
[52]	25	64.5 FSC	50	nd	25	50 mM L^−1^ phosphate buffer, 1% hydroxypropyl- β-CD, 3.3% isopropanol	2.5	cytosine, sophocarpine, matrine, lehmannine, sophoranol, oxymatrine, oxysophocarpine	200	nd
[53]	20	35 FSC	50	375	20	500 mM L^−1^ Tris-H_3_PO_4_ buffer, 50% (*v*/*v*) methanol, 2 mM L^−1^ HP-β-CD	2.5	sanguinarine, coptisine, chelerythrine, berberine, chelidonine, protopine, allocryptopine, stylopine	205	4.27 4.28 1.87 3.78 0.69 0.75 0.65 0.90
[54]	nd	nd FSC	nd	nd	nd	20 mM L^−1^ Na_2_HPO_4_, 10% methanol	7.0	berberine, jatrorrhizine, palmatine	nd	0.3 0.3
[55]	25	52 FSC	50	nd	30	100 mM L^−1^ ammonium acetate in methanol, acetonitrile, water (70/25/5; *v*/*v*/*v*) with 0.6% acetic acid	nd	(−)-caaverine, (+)-isoliensinine, (+)-norarmepavine, (−)-armepavine, (−)-nuciferine, (−)-nornuciferine, (+)-pronuciferine	225	1.53 1.28 0.82 0.55 0.45 1.16 1.28
[56]	30	48.5 FSC	75	nd	20	10 mM L^−1^ Na_2_HPO_4_, 6 mM L^−1^ Na_3_PO_4_, 15% methanol (*v*/*v*)	11.8	physcion, aloe-emodin, chrysophanol, emodin, aurantio-obtusin, rhein	254	0.94 0.42 0.33 0.43 1.40 1.03
[57]	15	35 FSC	50	365	25	20 mM L^−1^ borax buffer	10.5	HC, HMC, 7-GC	230	3.75 0.94 3.75
[58]	15	65 nd	50	375	RT	50 mM L^−1^ copper sulfate and 0.5% acetic acid (*v*/*v*)	4.5	L-phenylalanine, L-histidine, L-leucine, L-glutamic, L-proline	254	5.0 M 3.0 M 1.0 M 2.0 M 3.0 M

nd—no data, V—voltage (KV), L_cap_—length of capillary (cm), BGE—background electrolyte, ^—material of capillary: FSC—fused silica capillary, QC—quartz capillary, ID—inner diameter of capillary (µm), OD—outer diameter of capillary (µm), T—temperature (°C), RT—room temperature, λ—wavelength (nm), LOD—limit of detection (µg mL^−1^), *—reference method, β-CD—β-cyclodextrin, GL—7-*O*-α-L-rhamnopyranosyl-kaempferol-3-*O*-β-D-glucopyranoside, RH—7-*O*-α-L-rhamnopyranosyl-kaempferol-3-*O*-α-L-rhamnopyranoside, TTMF—5,7,4′-trihydroxy-6,3′,5′-trimethoxyflavone, HC—7-hydroxy-coumarin, HMC—7-hydroxy-8-methoxy-coumarin, 7-GC—7-*O*-β-D-glucosyl-coumarin.

**Table 3:** Application of capillary electrophoresis with electrochemical detection.

Ref	Instrumental Variables					Chemical Variables		Compounds	Method	LOD (µg mL^−1^)
	V (kV)	Lcap ^ (cm)	ID (µm)	OD (µm)	T (°C)	BGE	pH			
[60]	30	40 FSC	25	360	nd	50 mM L^−1^ borate buffer	9.2	neferine, liensinine, isoliensinine, rutin, hyperoside	CE-AD	0.67 µM 0.50 µM 0.62 µM 0.57 µM 0.84 µM
[25]	18	75 FSC	25	360	nd	30 mM L^−1^ borate, 40 mM L^−1^ phosphate	8.5	L-theanine, L-glutamine, sucrose, glucose, fructose, ascorbic acid, (−)-epigallocatechin gallate	CE-AD	0.53 µM 0.47 µM 0.24 µM 0.07 µM 0.53 µM 2.39 µM 0.86 µM
[59]	15.0	60 FSC	25	360	RT	50 mM L^−1^ KH_2_PO_4_ 250 mM L^−1^ Na_2_B_4_O_7_ and 0.20 mM L^−1^ β-CD	7.6	kaempferol, apigenin, rutin, ferulic acid, quercetin, luteolin	CE-AD	0.92 × 10^−4^ 0.79 × 10^−4^ 2.59 × 10^−4^ 1.90 × 10^−4^ 1.37 × 10^−4^ 1.13 × 10^−4^
[61]	19.5	60 FSC	25	370	nd	60 mM L^−1^ Na_2_B_4_O_7_, 120 mM L^−1^ NaH_2_PO_4_	8.8	catechin, rutin, hyperoside, quercetin, quercitrin	CE-ED	0.02 0.05 0.02 0.03 0.02
[62]	30.0	40 FSC	25	360	RT	7.5 mM L^−1^ NaH_2_PO_4_, 7.5 mM L^−1^ borax	6.0	isofraxidin, rutin, chlorogenic acid	CE-ED	0.10 µM 0.20 µM 0.15 µM
[63]	12.5	40 FSC	25	360	RT	20 mM L^−1^ phosphate buffer solution	10.0	aristolochic acid I, aristolochic acid II	CE-ED	0.04 µM 0.01 µM
[64]	16	75 FSC	25	nd	nd	50 mM L^−1^ borate buffer	9	genistin, genistein, rutin, kaempferol, quercetin	CE-ED	0.12 0.11 0.20 0.17 0.28

nd—no data, V—voltage (KV), Lcap—length of capillary (cm), BGE—background electrolyte, ^—material of capillary: FSC—fused silica capillary, ID—inner diameter of capillary (µm), OD—outer diameter of capillary (µm), T—temperature (°C), RT—room temperature, λ—wavelength (nm), LOD—limit of detection (µg mL^−1^), CE-AD—capillary electrophoresis with amperometric detector, CE-ED—capillary electrophoresis with electrochemical detection.

**Table 4:** Application of capillary electrophoresis with fluorescence detection.

Ref	Instrumental Variables					Chemical Variables		Compounds	λ (nm)	LOD (µM)
	V (kV)	Lcap ^ (cm)	ID (µm)	OD (µm)	T (°C)	BGE	pH			
[72]	21	80 FSC	50	360	23	30 mM L^−1^ sodium tetraborate, 20 mM L^−1^ OPA/2-ME	10.0	γ-aminobutyric acid, alanine	495	0.004 0.02
[73]	21	80 FSC	50	365	23	50 mM L^−1^ sodium tetraborate	10.0	γ-aminobutyric acid, alanine	495	700 800
[74]	15	60 FSC	75	365	nd	10 mM L^−1^ Na_2_B_4_O_7_	9.3	γ-aminobutyric acid, glycine, aspartic acid	410	360–28,300
[71]	25	50 FSC	50	nd	25	30 mM L^−1^ phosphate buffer	9.9	riboflavin	488	1.08 ng mL^−1^
[75]	18	55 FSC	100	nd	RT	water/acetonitrile (9:1, *v*/*v*), 10 mM L^−1^ borate buffer	9.6	riboflavin	474	300,0
[76]	10	30 FSC	75	nd	25	5% acetonitrile, 0.25% acetic acid, 35 mM L^−1^ ammonium acetate in methanol	nd	berberine, palmatine, jatrorrhizine	488	6.0 ng mL^−1^ 7.5 ng mL^−1^ 380 ng mL^−1^
[77]	25	50.2 QC	75	nd	30	5 mM borate buffer, 10 ^−4^ mM fluorescin sodium salt, 20% methanol	9.4	naringin, sophoricoside, esculin, genistein, isofraxidin, esculetin	488 520	20.2 5.0 7.5 7.5 6.8 9.7

nd—no data, V—voltage (KV), Lcap—length of capillary (cm), BGE—background electrolyte, ^—material of capillary: FSC—fused silica capillary, QC—quartz capillary, ID—inner diameter of capillary (µm), OD—outer diameter of capillary (µm), T—temperature (°C), RT—room temperature, λ—wavelength (nm), LOD—limit of detection (µg mL^−1^), OPA—*o*-phthaldialdehyde; 2-ME—2-mercaptoethanol.

**Table 5:** Application of micellar electrokinetic chromatography.

Ref	Instrumental Variables					Chemical Variables		Compounds	λ (nm)	LOD (µg mL^−1^)
	V (kV)	Lcap ^ (cm)	ID (µm)	OD (µm)	T (°C)	BGE	pH			
[78]	20	50 FSC	75	nd	25	15 mM L^−1^ borate, 40 mM L^−1^ phosphate, 15 mM L^−1^ SDS, 15% (*v*/*v*) acetonitrile, 7.5% (*v*/*v*) 2-propanol	nd	baicelin, baicalein, wogonin	280	1.79 1.19 0.78
[79]	15.0	8.5 nd	nd	nd	25	25mM L^−1^ borate– phosphate buffer, 25 mM L^-1^ HP-β-CD, 90 mM L^−1^ SDS	2.5	(−)-EGC; (−)-GCG; (−)-C; (−)-ECG; (−)-EGCG; (+)-C; (−)-EC; CAF; TB; (−)-GC; (+)-GC;	200	0.05 0.15 0.1 0.1 0.2 0.4 0.7
[80]	25	62 FSC	50	nd	40	50 mM L^−1^ borax, 25 mM SDS, 30% acetonitrile	6.75	kaempferol-3-*O*-glucoside, 6-methoxy-kaempferol 3-*O*-glucoside, hispidulin, quercetin 3-*O*-glucoside, patuletin-3-*O*-glucoside, quercetin 3-*O*-glucuronic acid, chlorogenic acid, 3,5-dicaffeoylquinic acid, 4,5-dicaffeoylquinic acid	254	≤4.5
[81]	30	64.5 nd	50	nd	27	50 mM L^−1^ sodium tetraborate, 60 mM L^−1^ SC, 20% methanol (*v*/*v*)	9.0	coumarin, scoparone, xanthotoxin, byakangelicin, isoscopoletin, esculin, isopimpinellin, bergapten, esculetin, phellopterin, xanthotoxol, umbelliferone	214	0.575 0.927 1.042 1.290 1.558 1.273 1.512 1.358 0.860 1.987 1.599 1.422
[82]	20	50.2 FSC	75	375	25	20 mM L^−1^ sodium borate, 20 mM Brij 35, acetonitrile 10%	8.5	lysine, phenylalanine, leucine, methionine, valine, theanine, histidine, γ-aminobutyric acid, threonine, alanine, serine, glycine, cysteine, glutamic acid, aspartic acid	nd	0.5 ng mL^−1^ 0.2 ng mL^−1^ 0.2 ng mL^−1^ 0.5 ng mL^−1^ 0.2 ng mL^−1^ 0.5 ng mL^−1^ 10 ng mL^−1^ 0.1 ng mL^−1^ 0.5 ng mL^−1^ 0.3 ng mL^−1^ 0.5 ng mL^−1^ 0.2 ng mL^−1^ 100 ng mL^−1^ 3 ng mL^−1^ 5 ng mL^−1^
[83]	20	50 FSC	75	375	25	10 mM L^−1^ sodium tetraborate, 20 mM β-CD, 20 mM SDS	9.2	serotonin, melatonin, tryptophan, indole-3-acetic acid	220	4.16 ng g^−1^ 0.79 ng g^−1^ 0.72 ng g^−1^ 0.55 ng g^−1^
[84]	30	48.5 FSC	8.5	nd	30	25 mM L^−1^ borate phosphate buffer, 65 mM L^−1^ SDS, 28 mM L^−1^ Heptakis (2,6-di-*O*-methyl)- β-CD	2.5	(−)-EGC; (−)-C; (−)-ECG; L-THE; D-THE; (−)-EGCG; (−)-EC; (+)-C; (−)-GCG;	220	0.1 0.2 0.2 0.2
[86]	30	60 FSC	50	nd	25	0.03 M L^−1^ borate buffer, 10% acetonitrile, 0.01 mM L^−1^ SDS	10.2	apigenin	390	0.48 µmol L^−1^

nd—no data; V—voltage (KV), L_cap_—length of capillary (cm), BGE—background electrolyte, ^—material of capillary: FSC—fused silica capillary, ID—inner diameter (µm), OD—outer diameter (µm), T—temperature (°C), RT—room temperature, λ—wavelength (nm), LOD—limit of detection (µg mL^−1^), SDS—sodium dodecyl sulfate; HP-β-CD—hydroxypropyl-β-cyclodextrin; (−)-EC—(−)-epicatechin; (−)-ECG—(−)-epicatechin gallate; (−)-EGC—(−)-epigallocatechin; (−)-EGCG—(−)-epigallocatechin gallate; (+)-C—(+)-catechin; (−)-C—(−)-catechin; (−)-GCG—(−)-gallocatechin gallate; (+)-GC—(+)-gallocatechin; (−)-GC—(−)-gallocatechin; CAF—caffeine; TB—theobromine; THE—theanine; nd—no data, OPA—*o*-phthaldialdehyde; 2-ME—2-mercaptoethanol.

**Table 6:** Process of separation of analytes with application capillary isotachophoresis.

Ref	Instrumental Variables					Chemical Variables		Compounds	λ (nm)	LOD (µg mL^−1^)
	V (kV)	Lcap ^ (cm)	ID (µm)	OD (µm)	T (°C)	BGE	pH			
[87]	nd	60 FEP	300	nd	nd	LE: 10 mM L^−1^ HCl, 0.02 M IMI, 0.2% HEC TE: 0.01 M HEPES	7.2 8.2	protocatechuic acid, caffeic acid, cinnamic acid, vanillic acid, coumaric acid, syringic acid, ferulic acid, chlorogenic acid	270	0.041 0.034 0.005 0.044 0.010 0.026 0.021 0.061
[88]	3	16 FEP	300	nd	nd	25 mM L^−1^ MOPSO, 50 mM L^−1^ Tris buffer, 40 mM L^−1^ H_3_BO_3_, 0.2% HEC	8.1	caffeic acid, rosmarinic acid, p-coumaric acid, chlorogenic acid, ferulic acid, quercitrin	320	0.018 0.027 0.030 0.032 0.020 0.035

nd—no data, V—voltage (KV), Lcap—length of capillary (cm), BGE—background electrolyte, ^—material of capillary: FEP—fluorinated ethylene-propylene (FEP) copolymer, ID—inner diameter (µm), OD—outer diameter (µm), T—temperature (°C), RT—room temperature, λ—wavelength (nm), LOD—limit of detection (µg mL^−1^), LE—leading electrolyte; TE—terminating electrolyte, IMI—imidazole, HEC—hydroxyethylcellulose, HEPES—4-(2-Hydroxyethyl)-1-piperazine ethanesulfonic acid, MOPSO—3-(*N*-morpholino)-2-hydroxypropanesulfonic acid.

**Table 7:** Process of separation of analytes with application capillary electrochromatography.

Ref	Instrumental Variables					Chemical Variables		Compounds	λ (nm)	LOD (µg mL^−1^)
	V (kV)	Lcap ^ (cm)	ID (µm)	OD (µm)	T (°C)	BGE	pH			
[93]	25	50 FSC	75	nd	25	50 mM L^−1^ phosphate buffer (pH 2.8) and 50% acetonitrile		herniarin, umbelliferone, caffeic acid, chlorogenic acid, apigenin, naringenin, apigenin-7-*O*-glucoside, luteolin, luteolin-7-*O*-glucoside, quercetin, rutin	337	35.0 35.0 35.0 35.0 35.0 35.0
[94]	10	33 FSC	75/100	375	20	acetate buffer	4.0	epigallocatechin, theophylline, catechin, epicatechin, caffeine	200	1.0 1.0 1.0
[92]	10	nd FSC	75	375	nd	10 mM L^−1^ ammonium formate	3.0	epicatechin, apigenin	260	50.0 ng 50.0 ng
[95]	−6	20 FSC	100	375	nd	10 mM L^−1^ ammonium acetate buffer and acetonitrile (50/50, *v*/*v*)	4.0	bergapten, imperatorin, osthole, 2′-acetylangelicin, oroselone, *O*-acetylcolumbianetin	320	2.5 5.0 1.0 2.5 2.5 2.5
[5]	−25	50 FSC	100	365	nd	30% acetonitrile and 1% ammonia aqueous solution	8.2	limonin, evodiamine, rutaecarpine	CEC-MS	3.10 0.63 0.15

nd—no data, V—voltage (KV), Lcap—length of capillary (cm), BGE—background electrolyte, ^—material of capillary: FSC—fused silica capillary, ID—inner diameter (µm), OD—outer diameter (µm), T—temperature (°C), RT—room temperature, λ—wavelength (nm), LOD—limit of detection (µg mL^−1^), LE—leading electrolyte; TE—terminating electrolyte.

**Table 8:** Process of separation of analytes with application capillary electrophoresis with MS detection.

Ref	Instrumental Variables					Chemical Variables		Compounds	LOD (fg)
	V (kV)	Lcap ^ (cm)	ID (µm)	OD (µm)	T (°C)	BGE	pH		
[96]	30	70 FSC	30	150	nd	1% acetic acid and methanol/water (1/1)	nd	coptisine, berberine, palmatine, jatrorrhizine	18.0 22.0 24.0 16.0
[97]	25	80 FSC	50	nd	25	200 mM L^−1^ boric acid and ammonia	9.0	chlorogenic acid, delphinidin-3-*O*-glucoside, cyaniding-3-*O*-rutinoside, cyaniding-3-*O*-sambubioside, delphinidin-3-sambubioside, Cy-3,5-*O*-diglucoside	nd

nd—no data, V—voltage (KV), Lcap—length of capillary (cm), BGE—background electrolyte, ^—material of capillary: FSC—fused silica capillary, ID—inner diameter (µm), OD—outer diameter (µm), T—temperature (°C), RT—room temperature, λ—wavelength (nm), LOD—limit of detection (µg mL^−1^), LE—leading electrolyte; TE—terminating electrolyte.

**Table 9:** PICOS (Population, Intervention, Comparison, Outcome, Study type).

	Inclusion Criteria	Exclusion Criteria
Population	raw material plant using in traditional medicine	garden and ornamental plants, spices, vegetables, and fruits
Intervention	use of CE method	other methods
Comparison	capillary electrophoresis vs. other methods	not applicable
Outcome	analysis of bioactive compounds (polyphenols, coumarins, vitamins, alkaloids)	different outcomes
Study type	original research articles, full articles, English language	review articles, reports, abstracts, articles with no quantitative information or details

## Abbreviations

A	apigenin
*p*A	*p*-coumaric acid
AA-I	aristolochic acid I
AA-II	aristolochic acid II
AAS	Atomic Absorption Spectrometry
AD	amperometric detection
ASC	ascorbic acid
ASP	aspartic acid
B	baicalin
BC	baicalein
BER	berberine
BGE	background electrolyte
BMIM-PF6	1-butyl-3-methyl imidazolium hexafluorophosphate
CA	carnosic acid
CAA	caffeic acid
CAF	caffeine
CAT	catechin
CCE	Chiral capillary electrophoresis
β-CD	β-cyclodextrin
CE	capillary electrophoresis
CE-AD	capillary electrophoresis with amperometric detector
CEC	capillary electrochromatography
CE-C^4^D	capillary electrophoresis with capacitively coupled contactless conductivity detection
CE-ED	capillary electrophoresis with electrochemical detection
CE-LIF	capillary electrophoresis with laser-induced fluorescence detection
CGE	capillary gel electrophoresis
CIEF	capillary isoelectric focusing
CITA	citric acid
CITP	capillary isotachophoresis
CLA	chlorogenic acid
CSEID	class-selective electrochemical index determination
CZE	capillary zone electrophoresis
DHB	3,4-dihydroxybenzaldehyde
3,4-DHBA	3,4-dihydroxybenzoic acid
EC	epicatechin
ECG	epicatechin gallate
EGC	epigallocatechin
EGCG	(−)-epigallocatechin gallate
EOF	electroosmotic flow
ESI	electrospray ionization
FA	ferulic acid
FRU	fructose
FSC	fused silica capillary
G	guanosine
GABA	γ-aminobutyric acid
GC	Gas Chromatography
7-GC	7-*O*-β-D-glucosyl-coumarin
GEMBE	gradient elution moving boundary electrophoresis
GL	7-*O*-α-L-rhamnopyranosyl-kaempferol-3-*O*-β-D-glucopyranoside
GLU	glucose
L—GLU	L—glutamine
GLY	glycine
H	hyperoside
HA	homogentisic acid
HC	7-hydroxy-coumarin
HEC	hydroxyethylcellulose
HEPES	4-(2-Hydroxyethyl)-1-piperazine ethanesulfonic acid
HMC	7-hydroxy-8-methoxy-coumarin
HP-β-CD	hydroxypropyl-β-cyclodextrin
HPLC	High-Performance Liquid Chromatography
ISOF	isofraxadin
IAD	individual antioxidant determination
ICP-AES	Inductively Coupled Plasma-Atomic Emission Spectrometry
ICP-OES	Inductively Coupled Plasma Optical Emission Spectrometry
ICP-MS	Inductively Coupled Plasma Mass Spectrometry
ID	inner diameter of capillary (µm)
IMI	imidazole
JATR	jatrorrhizine
K	kaempferol
Lcap	length of capillary (cm)
LA	lactic acid
LE	leading electrolyte
LED-IF	light-emitting diode induced fluorescence detection
LIF	laser induced fluorescence
LOD	limit of detection
LUT	luteolin
M	methionine
MA	malic acid
2-ME	2-mercaptoethanol
MEEKC	microemulsion electrokinetic capillary chromatography
MEKC	micellar electrokinetic chromatography
MES	2-(*N*-morpholino)ethanesulfonic acid
MFAS	microfluidic analytical system
MOPSO	3-(*N*-morpholino)-2-hydroxypropanesulfonic acid
MS	mass spectrometry
NACE	non-aqueous capillary electrophoresis
OD	outer diameter of capillary (µm)
OPA	*o*-phthaldialdehyde
OTC	open-tubular columns
PAL	palmitine
PEO	ply(ethylene oxide)
PICOS	Population, Intervention, Comparison, Outcome, Study type
SDS	sodium dodecyl sulfate
SC	sodium salt
TE	terminating electrolyte
Q	quercetin
QC	quartz capillary
QU	quercitrin
R	rutin
RA	rosmarinic acid
RH	7-*O*-α-L-rhamnopyranosyl-kaempferol-3-*O*-α-L-rhamnopyranoside
RT	room temperature
SA	succinic acid
SPE	solid phase extraction
D-Th	D-Theanine
T	temperature (°C)
TA	tartaric acid
TB	theobromine
L-THE	L-theanine
TLC	Tin Layer Chromatography
TTMF	5,7,4′-trihydroxy-6,3′,5′-trimethoxyflavone
V	voltage (kV)
W	wogonin
λ	wavelength (nm)
